# Pituitary and Optic Tract Lesions Masquerading As Glaucoma: Highlighting Diagnostic Challenges

**DOI:** 10.7759/cureus.93227

**Published:** 2025-09-25

**Authors:** Priti Singh, Samendra Karkhur, Vidhya Verma, Saroj Gupta, Rama Tulasi Siri Duddumpudi, Radhika Agarwal

**Affiliations:** 1 Ophthalmology, All India Institute of Medical Sciences, Bhopal, IND

**Keywords:** compressive optic neuropathy, optical coherence tomography (oct), optic tract lesion, pituitary macroadenoma, visual field defects

## Abstract

Purpose

This study's purpose is to highlight the diagnostic pitfalls in distinguishing glaucomatous optic neuropathy from compressive optic neuropathies caused by intracranial lesions, particularly pituitary macroadenomas.

Methodology

This research was a retrospective case series of six patients who were originally diagnosed or suspected to have glaucomatous optic neuropathy but later revealed intracranial lesions on neuroimaging.

Results

All patients had optic disc cupping and/or pallor with visual field defects (VFDs) overlapping with glaucomatous patterns. However, atypical features such as VFDs respecting the vertical meridian, homonymous hemianopia, optic disc pallor disproportionate to cupping, and poor correlation between structure and function raised suspicion for non-glaucomatous causes. Neuroimaging revealed pituitary macroadenoma in four patients, parasellar meningioma in one, and an optic tract lesion in another.

Conclusion

Compressive optic neuropathies may closely mimic glaucoma, resulting in delayed diagnosis and inappropriate treatment. Careful attention to atypical field patterns and disc findings, combined with timely neuroimaging, is crucial for avoiding irreversible vision loss.

## Introduction

Pituitary macroadenomas and other sellar or suprasellar lesions are relatively common intracranial tumors, with pituitary adenomas accounting for approximately 10-15% of all primary brain tumors [1]. When large enough, these lesions can compress the optic chiasm, optic nerves, or optic tracts, producing characteristic patterns of visual field loss. In many cases, these deficits-particularly in the early stages-may mimic primary open-angle glaucoma (POAG) or normal-tension glaucoma (NTG), leading to misdiagnosis and delayed treatment [2]. The overlap in clinical presentation is primarily due to optic disc cupping, visual field defects (VFDs), and chronic, insidious visual loss, features common to both glaucomatous and compressive optic neuropathies [3].

The risk of diagnostic error is especially high when VFDs are asymmetric or when intraocular pressures (IOPs) are within normal limits, prompting a label of NTG [1,4]. However, certain clues, such as VFDs respecting the vertical meridian, optic disc pallor disproportionate to the degree of cupping, and poor correlation between structural (optic nerve head) and functional (visual field) findings, should raise suspicion for a non-glaucomatous etiology [5,6]. In addition, optical coherence tomography (OCT) and OCT angiography (OCTA) have shown utility in differentiating glaucomatous from compressive optic neuropathies by revealing distinct patterns of retinal nerve fiber layer (RNFL) loss and microvascular compromise [7].

Despite these distinguishing features, the literature documents multiple cases in which patients with pituitary macroadenomas or other compressive lesions were treated for glaucoma for prolonged periods before the correct diagnosis was made [3,8]. Such delays can have serious consequences, as irreversible vision loss may occur even after successful tumor removal or medical management if intervention is not timely [2]. Therefore, neuroimaging - preferably magnetic resonance imaging (MRI) of the brain and orbit - should be considered in all patients with atypical glaucomatous presentations, especially when red-flag features are present [1].

In this article, we present six patients initially suspected or treated for glaucoma, who were later diagnosed with compressive optic neuropathy based on CT/MRI findings. Through these cases, we emphasize the importance of early recognition of atypical patterns and prompt neuroimaging to prevent unnecessary treatment, facilitate targeted management, and preserve vision.

## Materials and methods

Study design and setting

We conducted a retrospective, descriptive case series at a tertiary care ophthalmology center, evaluating consecutive patients who were initially labeled as glaucoma or glaucoma suspects but were later found to harbor intracranial lesions on neuroimaging. The series comprised six patients seen over a six-month accrual period.

Ethical approval and consent

The study protocol was approved by the Institutional Human Ethics Committee (Institutional Review Board (IRB) approval IHEC-LOP/2025/IP051). The research adhered to the tenets of the Declaration of Helsinki. Written informed consent for clinical care and publication of de-identified images was obtained or waived as per IRB guidance.

Patient identification and eligibility

Inclusion criteria were (1) prior clinical diagnosis or referral as glaucoma/glaucoma suspect; (2) presence of optic disc cupping and/or pallor with corresponding visual field changes on standard automated perimetry; and (3) subsequent confirmation of an intracranial lesion (e.g., pituitary macroadenoma, parasellar meningioma, optic tract lesion) on neuroimaging with clinico-radiologic correlation. Exclusion criteria were (1) incomplete records precluding confirmation of the visual field or imaging findings; (2) media opacities or ocular comorbidities (e.g., dense cataract, corneal scar) that rendered perimetry/OCT unreliable; (3) known non-compressive optic neuropathies (toxic, nutritional, hereditary) or acute optic neuritis. Although the study was retrospective, we applied strict predefined exclusion criteria to rule out other causes of optic neuropathy. Specifically, cases with a documented history or laboratory/imaging evidence suggestive of toxic, nutritional, hereditary, or demyelinating optic neuropathies were excluded. The medical records included systemic evaluations (complete blood count (CBC), erythrocyte sedimentation rate (ESR), antinuclear antibody (ANA)/anti-neutrophil cytoplasmic antibody (ANCA), vitamin levels when indicated), CSF studies in selected cases, and neuroimaging findings, all of which supported a compressive etiology rather than alternative diagnoses. Thus, the exclusion was based not on past history alone but on a combination of clinical documentation, laboratory data, and radiological correlation, and (4) prior intracranial surgery or radiotherapy altering neuro-anatomy before ophthalmic assessment.

Data collection and variables

Data were abstracted from the electronic medical record by two investigators using a standardized template and cross-checked for accuracy. Variables captured included demographics; presenting symptoms and duration; best-corrected visual acuity (BCVA); IOP; central corneal thickness (CCT); pupillary responses, including relative afferent pupillary defect (RAPD); color vision; slit-lamp and dilated fundus findings (cup-disc ratio, pallor vs. cupping); perimetry indices; OCT parameters (RNFL and macular ganglion cell-inner plexiform layer (GCIPL)); neuroimaging characteristics; final diagnosis; and downstream management (neurosurgery/endocrinology/neurology referrals).

Ophthalmic examination protocols

All examinations were performed by trained glaucoma and neuro-ophthalmology faculty. While more than one examiner was involved, a uniform departmental protocol was followed, including Goldmann applanation tonometry for IOP, optical pachymetry for CCT, slit-lamp biomicroscopy with 78D/90D lens for disc assessment, and standardized use of the same Humphrey Visual Field (HVF) Analyzer and Cirrus OCT (Carl Zeiss Meditec, Jena, Germany). Documentation was cross-verified in the electronic medical record to ensure consistency across cases. BCVA was measured using an illuminated Snellen chart at 6 m with habitual correction; IOP was assessed by Goldmann applanation tonometry (Haag-Streit AG, Köniz, Switzerland) after topical anesthesia, and CCT was measured by optical pachymetry (Haag-Streit AG, Köniz, Switzerland). Slit-lamp biomicroscopy (Haag-Streit AG, Köniz, Switzerland) and dilated fundus examination using 78D/90D lenses (Volk Optical Inc., Mentor, OH) documented disc morphology, with emphasis on pallor versus cupping and neuro-retinal rim contour. OCT (Carl Zeiss Meditec AG, a division of Carl Zeiss AG, Jena, Germany) and HVF Analyzer (Carl Zeiss Meditec AG, a division of Carl Zeiss AG, Jena, Germany) were used for structural and functional assessment, respectively.

Standard automated perimetry

All testable eyes underwent HVF testing using either the 30-2 or 24-2 testing pattern with the SITA Standard strategy. Reliability criteria were predefined (fixation losses ≤20%, false positives ≤15%, false negatives interpreted in clinical context). Key global indices (MD, PSD, VFI) and Glaucoma Hemifield Test (GHT) status were recorded [9]. Field patterns were categorized a priori as glaucomatous (arcuate/nasal step/altitudinal respecting the horizontal meridian) or atypical/neurologic (bitemporal, homonymous, or incongruous hemianopic patterns respecting the vertical meridian).

OCT imaging

Cirrus spectral-domain OCT was used to acquire peripapillary RNFL and macular GCIPL scans following the manufacturer’s signal strength and centration criteria. Scans with motion artefacts, segmentation errors, or signal strength below device-recommended thresholds were repeated. Patterns were labeled glaucomatous (sectoral arcuate RNFL loss with structure-function concordance) versus compressive (diffuse or temporally predominant RNFL/GCIPL thinning with poor structure-function concordance), based on pre-specified definitions.

Neuroimaging protocol

These cases illustrate how subtle but important red-flag signs, such as optic disc pallor disproportionate to cupping or VFDs respecting the vertical meridian, may be overlooked in routine practice, leading to initial mislabeling as glaucoma and underscoring the need for heightened clinical vigilance. Patients with red-flag findings (pallor > cupping, vertical-meridian-respecting VFDs, poor structure-function correlation, young age, rapid progression, or discordant IOP/CCT profile) underwent neuroimaging. Preferred imaging was MRI of the brain and orbits with contrast using standard sequences (T1-weighted pre-/post-contrast, T2, and FLAIR) to assess the chiasm, optic nerves, and tracts. Non-contrast computed tomography (NCCT) was used when MRI was contraindicated/unavailable. Lesions were classified (e.g., pituitary macroadenoma, parasellar meningioma, optic tract lesion) by a neuroradiologist, and final diagnoses were established by clinico-radiologic correlation.

Outcomes and definitions

The primary outcome was identification of a compressive optic neuropathy masquerading as glaucoma among referred glaucoma suspects. Secondary outcomes included description of characteristic HVF and OCT patterns, and subsequent changes in management (cessation of anti-glaucoma therapy; neurosurgical/endocrine/neurology referral). Vertical-meridian-respecting defects (bitemporal or homonymous/incongruous hemianopia) and pallor disproportionate to cupping were pre-specified as “red-flag” indicators.

Image preparation and figure labeling

All images were de-identified before analysis. For publication, extraneous borders and patient ID headers/footers were cropped, and multi-panel figures were arranged with sublabels (A, B, C, …) positioned along the left margin. Legends explicitly define each subpanel (e.g., A: HVF; B: OCT RNFL/GCIPL).

Statistical analysis

Given the small, descriptive nature of this series, analyses were limited to descriptive statistics only. No hypothesis testing or regression modeling was performed. Tables summarize demographics/clinical triggers for neuroimaging, perimetry/OCT patterns, and neuroimaging-guided final diagnoses and actions.

## Results

Case series: intracranial lesions mimicking glaucoma

Case 1

**Figure 1:**
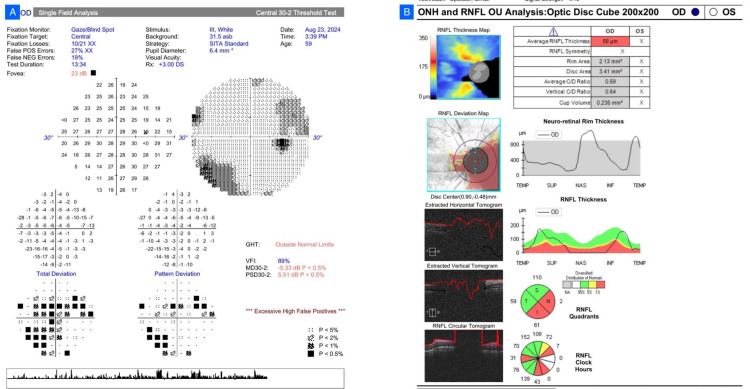
Case 1. (A) Humphrey Visual Field (HVF) 30-2 of the right eye showing a moderate defect (mean deviation (MD) -5.33 dB; pattern standard deviation (PSD) 5.51 dB) with Glaucoma Hemifield Test (GHT) outside normal limits. (B) Optical coherence tomography (OCT) retinal nerve fiber layer (RNFL) scan demonstrating temporal thinning corresponding to the visual field defect.

A 59-year-old female, with no family history of glaucoma, complained of reduced vision (worse in the left eye). BCVA was 6/18 in the right and perception of light with inappropriate projection in the left. Bilaterally, IOP was 24 mmHg with elevated CCT. There was a relative afferent pupillary defect in the left eye. Fundus inspection revealed pallor of the optic discs bilaterally, more severe on the left, which had already resulted in a false diagnosis of glaucoma and the start of topical Timolol 0.5% twice a day. HVF (30-2 SITA Standard) was possible in the right eye alone, with a moderate defect (MD -5.33 dB, PSD 5.51 dB) and GHT outside normal limits, but affected by high false positives. The field defect, along with corresponding temporal RNFL thinning, was more suggestive of compressive optic neuropathy over glaucoma. OCT established temporal RNFL loss (Figure 1). Neuroimaging (NCCT brain and orbit) revealed a mildly hyperdense left parasellar lesion, which was consistent with meningioma. The patient was referred to endocrinology and neurosurgery and is currently under follow-up.

Case 2

**Figure 2:**
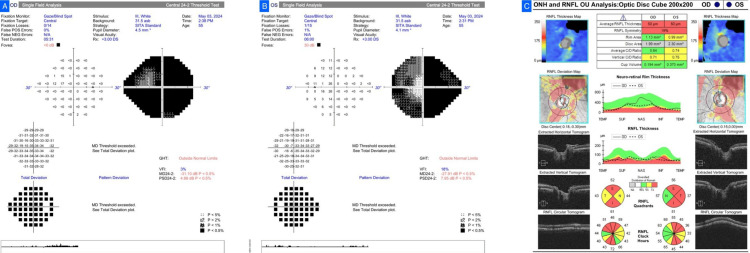
Case 2. (A, B) Humphrey Visual Field (HVF) 24-2 SITA Standard demonstrating bilateral temporal hemianopia respecting the vertical meridian. (C) Optical coherence tomography (OCT) retinal nerve fiber layer (RNFL) scan showing bilateral temporal thinning, more pronounced in the left eye.

A 55-year-old man with no family history of glaucoma presented with bilateral vision loss over three months. BCVA was 6/24 in the right eye and 6/9 in the left with normal IOPs. Bilateral optic disc pallor was seen on fundus examination, not characteristic of glaucomatous cupping. NTG had been previously diagnosed, and he was on topical bedtime Travoprost. HVF (24-2 SITA Standard) testing showed bilateral temporal hemianopia respecting the vertical meridian, highly suggestive of a chiasmal lesion. OCT showed bilateral thinning of RNFL (more in the left eye), specifically temporal (Figure 2). The NCCT brain showed a lobulated isodense sellar lesion with parasellar and suprasellar extension, typical of a pituitary macroadenoma. Glaucoma treatment was discontinued, and he was referred to neurosurgery and endocrinology for follow-up.

Case 3

**Figure 3:**
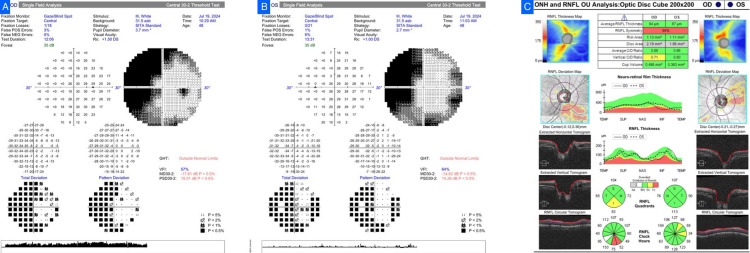
Case 3. (A, B) Humphrey Visual Field (HVF) 30-2 demonstrating advanced bilateral visual field loss with a relatively symmetric left-sided homonymous hemianopia. (C) Optical coherence tomography (OCT) retinal nerve fiber layer (RNFL) scan showing diffuse symmetric thinning in both eyes.

A 48-year-old female patient with rheumatic heart disease and iron deficiency anemia presented for glaucoma examination with headaches and field defects. BCVA was 6/12 in each eye, with normal IOP. Prior fundus records had documented cup-disc ratios of 0.5-0.6 bilaterally, with mild temporal pallor and tortuosity of vessels. She received Brimonidine twice daily for assumed NTG. HVF (30-2) revealed severe bilateral loss with relatively symmetrical left homonymous hemianopia, more typical for compressive optic neuropathy than glaucoma. OCT also showed diffuse symmetric thinning of the RNFL (Figure 3). MRI showed a lesion of the right optic tract with T2/fluid-attenuated inversion recovery (FLAIR) hyperintensity. She was referred to neurology for further evaluation and treatment.

Case 4

**Figure 4:**
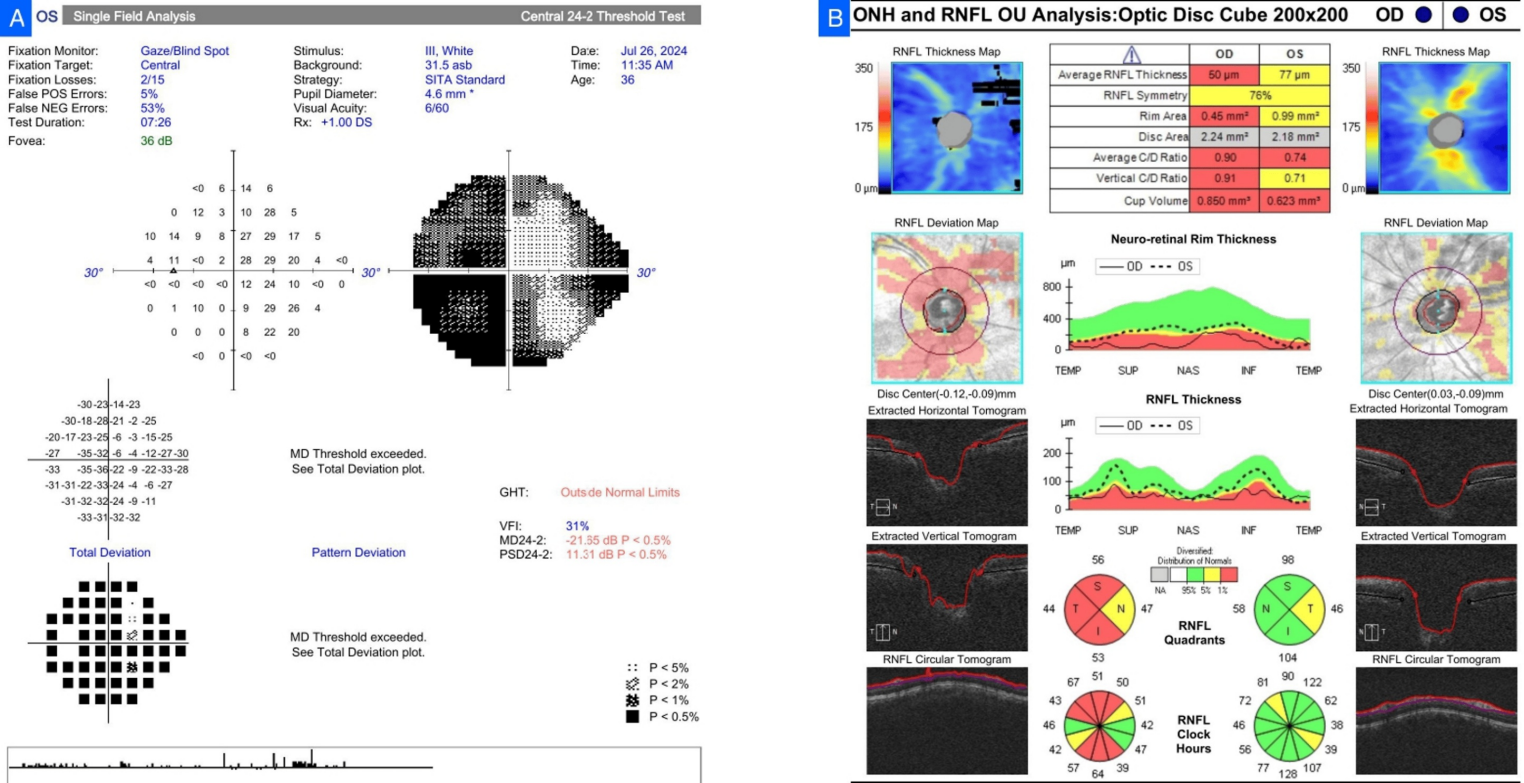
Case 4. (A) Humphrey Visual Field (HVF) of the left eye showing advanced generalized field loss (mean deviation (MD) -21.65 dB; visual field index (VFI) 31%). (B) Optical coherence tomography (OCT) retinal nerve fiber layer (RNFL) scan demonstrating diffuse thinning with optic disc pallor exceeding cupping.

A 36-year-old female patient complained of progressive loss of vision, more in the right eye, with intermittent headache. BCVA was hand movements near the face in the right eye and 6/12 in the left. Relative afferent pupillary defect was detected on the right. IOP was normal, along with color vision. Fundus revealed a pale disc with CDR of 0.7 in the right and 0.6 in the left. She was provisionally diagnosed with glaucoma by her referrer. HVF testing of the left eye demonstrated advanced generalized loss (MD -21.65 dB, VFI 31%) with diminished reliability, which showed a hemifield defect in favor of compressive neuropathy; the right eye could not be tested. OCT demonstrated diffuse thinning of the RNFL, with pallor more than cupping, again in favor of a compressive lesion (Figure 4). Contrast MRI showed a moderately enhancing sellar-suprasellar mass that is pressing on the optic chiasm, with encasement of the right optic nerve and surrounding vessels, a classic appearance of pituitary macroadenoma. She was referred to endocrinology and neurosurgery.

Case 5

**Figure 5:**
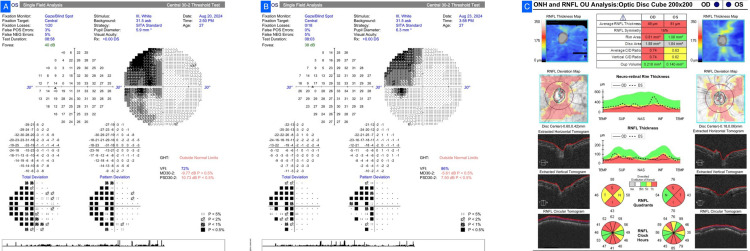
Case 5. (A) Humphrey Visual Field (HVF) 30-2 showing an incongruous left homonymous hemianopia. (B) Optical coherence tomography (OCT) retinal nerve fiber layer (RNFL) scan demonstrating diffuse thinning, more prominent in the temporal and superior quadrants, correlating with the visual field defect.

A female patient aged 27 years who had headaches and poor vision was referred for glaucoma assessment. The BCVA was 6/12 in the right eye and 6/24 in the left, with normal IOP. Fundus records were 0.5-0.6 CDR with temporal pallor, initially suggesting glaucoma. HVF (30-2) showed incongruous left hemianopia, a pattern more suggestive of post-chiasmal compressive optic neuropathy. OCT revealed diffuse thinning of RNFL, most prominent temporally and superiorly (Figure 5). MRI showed T2 hyperintensity and thinning of the right optic tract, extending posterior to the chiasm, with minimal atrophy of both optic nerves-findings consistent with a right optic tract lesion. She was referred to neurology for further care.

Case 6

**Figure 6:**
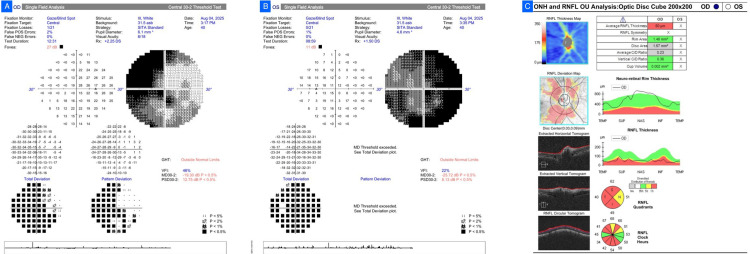
Case 6. (A, B) Humphrey Visual Field (HVF) 30-2 showing bilateral temporal hemianopia respecting the vertical meridian. (C) Optical coherence tomography (OCT) retinal nerve fiber layer (RNFL) scan showing temporal thinning in the right eye; the left eye could not be assessed due to poor fixation.

A 40-year-old man, without a family history of glaucoma, was seen with three years of bilateral visual impairment and a history of head trauma. BCVA was 6/36 in the right eye and 2/60 in the left. IOPs were normal. Fundoscopy showed bilateral optic disc pallor without characteristic glaucomatous cupping. He had been diagnosed previously as NTG. HVF (30-2 SITA Standard) revealed bilateral temporal hemianopia with respect to the vertical meridian, very suggestive of a chiasmal lesion. Right eye OCT showed temporal RNFL thinning, in keeping with optic neuropathy; left eye was not assessable because of poor fixation (Figure 6). MRI showed a lobulated sellar mass with suprasellar and parasellar extension and heterogeneous enhancement, in keeping with pituitary macroadenoma. Anti-glaucoma medication was stopped, and he was referred to neurosurgery and endocrinology. Table 1 has demographic details, clinical presentation, initial glaucoma suspicion, and reasons to consider non-neurological etiology.

**Table 1:** Demographic details, clinical presentation, initial glaucoma suspicion, and reasons to consider non-neurological etiology (e.g., pallor > cupping, raised CCT). CCT: central corneal thickness; CDR: cup-to-disc ratio; DOV: dimness of vision; IOP: intraocular pressure; NTG: normal-tension glaucoma

Case	Age/Sex	Key Symptoms	Reason Initially Diagnosed As Glaucoma	Reason to Suspect Non-glaucomatous Etiology	Duration/Progression of Symptoms
1	59/F	DOV OS > OD	Elevated IOP with disc pallor; on timolol	Pallor > cupping; corrected IOP lower with raised CCT	~3 months; subacute, progressive
2	55/M	Bilateral DOV × 3 months	NTG diagnosis elsewhere; normal IOP	Pallor > cupping	3 months; gradual progression
3	48/F	Headache, scotomas	NTG label (CDR 0.5-0.6 with pallor)	Pallor more than cupping	~6 months; insidious progression
4	36/F	DOV OU, intermittent headache	Large CDR, referred as glaucoma suspect	Pallor disproportionate to cupping	~1 year; slow progression
5	27/F	DOV, headache	Suspected glaucoma (CDR 0.5-0.6 with temporal pallor)	Temporal pallor > cupping; normal IOP	~8 months; gradual progression
6	40/M	Bilateral DOV × three years, trauma history	NTG diagnosis elsewhere; normal IOP	Pallor > cupping; positive trauma history	Three years; chronic

## Discussion

Clinical red flags in differentiation

The dissociation between disc pallor and cupping is an important clinical red flag. In glaucomatous optic neuropathy, the degree of pallor is usually proportional to the amount of cupping, since axonal loss occurs along the RNFL trajectories with corresponding excavation of the optic nerve head. In contrast, in compressive or neurological optic neuropathies, retrograde axonal degeneration leads to diffuse loss of ganglion cells and neuroretinal rim pallor, often with minimal or absent cupping. This mismatch - pallor exceeding cupping - should always raise suspicion of a non-glaucomatous etiology. Prior studies have consistently highlighted this discordance as a key differentiator: Gupta et al. [5] demonstrated that functional pituitary tumors may present with marked pallor despite modest cupping, and Senthil et al. [10] emphasized that disproportionate pallor, particularly in association with vertical-meridian-respecting field defects, warrants prompt neuroimaging to rule out compressive lesions. Recognizing this distinction can prevent unnecessary glaucoma treatment and facilitate the timely identification of vision-threatening intracranial pathology. In this series, all six patients presented with optic disc cupping and/or pallor along with VFDs that initially suggested glaucoma. However, atypical features-such as VFDs respecting the vertical meridian and homonymous hemianopia-were diagnostic red flags prompting neuroimaging. These findings are well documented in compressive optic neuropathies, most commonly pituitary macroadenomas and parasellar lesions [1,6,8]. Table 2 shows the HVF and OCT findings of the included cases.

**Table 2:** Humphrey Visual Field (HVF) and optical coherence tomography (OCT) findings of included cases. GCC: ganglion cell complex; GCIPL: ganglion cell–inner plexiform layer; MD: mean deviation; RNFL: retinal nerve fiber layer

Case	HVF Pattern	OCT Findings
1	Moderate defect, temporal RNFL thinning	Temporal RNFL thinning, GCIPL loss suggestive of compressive etiology
2	Bilateral temporal hemianopia	Thinning of temporal RNFL and GCIPL OU
3	Left homonymous hemianopia	RNFL/GCC loss predominantly contralateral to tract lesion
4	OS: advanced generalized loss (MD -21.65 dB; VFI 31%)	Bitemporal RNFL thinning, macular GCIPL loss
5	Incongruous left homonymous hemianopia	RNFL thinning in temporal quadrants with corresponding GCIPL loss
6	Bilateral temporal hemianopia	Bitemporal RNFL and GCIPL thinning

Distinguishing these entities requires correlation of structure and function. In glaucoma, optic disc pallor is generally proportional to cupping, whereas in compressive optic neuropathy, pallor often exceeds cupping [5,10]. Similarly, glaucomatous field defects typically follow RNFL trajectories and respect the horizontal meridian, while chiasmal or retrochiasmal lesions respect the vertical meridian [2,3,11].

Misdiagnosis and clinical consequences

Multiple reports document patients with pituitary lesions treated for glaucoma for prolonged periods before diagnosis was corrected [1,4,6,7]. Thinley et al. described a case of pituitary apoplexy misdiagnosed as NTG [4], while Cheng et al. highlighted a macroadenoma with optic cupping mimicking glaucoma [7]. Such misdiagnosis leads to unnecessary topical therapy and its adverse effects, without visual benefit [5,10]. Gupta et al. even reported that functional pituitary tumors may elevate IOP, complicating differentiation [5].

Importance of neuroimaging

Timely neuroimaging is critical. Delays can result in irreversible visual loss despite effective surgical or medical management [6,12,13]. Importantly, not all pituitary adenomas present with the “classic” bitemporal hemianopia; atypical field patterns are common [11]. Therefore, neuroimaging should be considered in patients with pallor > cupping, vertical meridian-respecting defects, or poor structure-function correlation [10,14]. Table 3 shows the Neuroimaging features, final diagnoses, and subsequent management decisions.

**Table 3:** Neuroimaging features, final diagnoses, and subsequent management decisions.

Case	MRI Finding	Final Diagnosis	Action Taken
1	Left parasellar lesion, probable meningioma	Compressive optic neuropathy (meningioma)	Anti-glaucoma meds stopped; referred to endocrinology and neurosurgery
2	Sellar lobulated mass, suprasellar extension → pituitary macroadenoma	Pituitary macroadenoma (chiasmal compression)	Anti-glaucoma meds stopped; referred to endocrinology and neurosurgery
3	Right optic tract T2/fluid-attenuated inversion recovery (FLAIR) hyperintensity	Right optic tract lesion	Referred to neurology
4	Enhancing sellar-suprasellar lesion, chiasmal compression	Pituitary macroadenoma	Referred to neurosurgery and endocrinology
5	Right optic tract hyperintensity extending posterior to chiasm	Right optic tract lesion	Referred to neurology
6	Lobulated sellar mass with suprasellar/parasellar extension	Pituitary macroadenoma	Anti-glaucoma meds stopped; referred to neurosurgery and endocrinology

Structural and vascular imaging correlates

OCT findings in compressive lesions often reveal diffuse or temporal RNFL thinning, while glaucoma causes localized arcuate damage [8,15]. GCIPL thickness has been shown to predict visual recovery after decompression [16]. OCTA further enhances differentiation: compressive neuropathies display diffuse vascular dropout compared to the sectoral loss of glaucoma [8,17]. Novel indices, such as the macular naso-temporal ratio, have also demonstrated utility in distinguishing glaucoma from chiasmal compression [17].

Beyond local changes, evidence supports retrograde trans-synaptic degeneration of retinal ganglion cells in chiasmal and retrochiasmal lesions [18-21]. Such degeneration explains the more widespread RNFL/GCIPL loss in compressive neuropathies compared to localized glaucomatous patterns [15,22].

Visual outcomes and prognostic factors

Surgical decompression through endoscopic endonasal approaches often improves vision, but outcomes depend on preoperative duration and severity [13]. Preserved GCIPL thickness and shorter symptom duration predict better recovery [16,23]. In our series, earlier referrals correlated with improved postoperative outcomes, in line with previous studies [23].

Emerging role of artificial intelligence

AI and machine learning hold promise in flagging atypical glaucoma suspects. Algorithms analyzing visual field and OCT data can detect suspicious patterns suggestive of compressive lesions [17,24]. Early work in neuro-ophthalmology highlights AI’s potential to standardize decision-making and reduce diagnostic delays [24]. Establishing multicentric registries of misdiagnosed cases could further improve awareness and provide real-world prevalence data [10]. Table 4 shows clinical clues differentiating glaucoma vs. compressive optic neuropathy. Table 5 shows reports of pituitary lesions misdiagnosed as glaucoma. Table 6 depicts imaging modalities for differentiation (from recent literature).

**Table 4:** Clinical clues differentiating glaucoma vs. compressive optic neuropathy. RNFL: retinal nerve fiber layer

Feature	Glaucoma	Compressive Optic Neuropathy	References
Optic disc changes	Pallor proportional to cupping	Pallor > cupping	[5,10]
Visual field defects	Follow RNFL trajectory; respect horizontal meridian	Respect vertical meridian; bitemporal/hemianopic patterns	[2,3,11]
Intraocular pressure (IOP)	Often raised	Usually normal; can be elevated in functional pituitary tumors	[5]
Structure-function correlation	Good correlation	Often poor correlation	[6,9,14]

**Table 5:** Reports of pituitary lesions misdiagnosed as glaucoma. IOP: intraocular pressure; NTG: normal-tension glaucoma

Author/Year	Case Details	Misdiagnosis	Correct Diagnosis	Key Learning Point
Osaguona et al., 2016 [1]	Adult male, NTG diagnosis	Glaucoma	Pituitary adenoma	Importance of neuroimaging
Thinley et al., 2024 [4]	Pituitary apoplexy	NTG	Pituitary macroadenoma	Acute presentation can mimic NTG
Gupta et al., 2014 [5]	Functional pituitary tumors	Primary glaucoma	Functional pituitary tumors	IOP can be elevated in compressive pathology
Cheng et al., 2023 [7]	Macroadenoma with cupping	NTG	Pituitary macroadenoma	Disc cupping does not exclude compressive lesion

**Table 6:** Imaging modalities for differentiation (from recent literature). OCT: optical coherence tomography; OCTA: optical coherence tomography angiography; RNFL: retinal nerve fiber layer

Modality	Findings in Glaucoma	Findings in Compressive Lesions	References
OCT (RNFL)	Localized arcuate thinning	Diffuse or temporal thinning	[8,16]
GCIPL	Sectoral loss	Diffuse thinning; predictive of visual recovery	[17,23]
OCTA	Sectoral capillary dropout	Diffuse capillary dropout	[8,18]
Advanced OCT indices	N/A	Macular naso-temporal ratio useful for differentiation	[18]
MRI	Usually normal	Sellar/parasellar/chiasmal lesion	[6,12,13]

Study limitations

This study has limitations. The small sample size (n = 6) limits generalizability. Being a single-center case series, it lacks long-term follow-up on postoperative outcomes. OCTA and other advanced modalities were not consistently available, restricting structural-vascular correlation. Referral bias may have also influenced case selection, as only atypical glaucoma suspects were included. Nonetheless, the series underscores critical diagnostic red flags and the need for integrated multimodal imaging and neuroimaging in glaucoma practice.

## Conclusions

This research case series underscores the critical importance of considering compressive optic neuropathies in the differential diagnosis of patients with optic disc cupping and VFDs that deviate from classical glaucomatous patterns. All six patients were initially suspected or treated for glaucoma, yet careful attention to atypical features, such as VFDs respecting the vertical meridian, optic disc pallor disproportionate to cupping, and poor structure, function correlation, prompted neuroimaging that revealed sight-threatening intracranial lesions. In our series, four patients had pituitary macroadenomas, one had a parasellar meningioma, and another had an optic tract lesion. Timely recognition of these red flags not only averted unnecessary and ineffective glaucoma therapy but also facilitated targeted neurosurgical and endocrine interventions, potentially preserving remaining vision. These findings highlight that a vigilant, pattern-based approach to visual field interpretation, coupled with judicious use of neuroimaging, can decisively alter patient outcomes and prevent irreversible visual disability.
